# Clinical Value on Combined Detection of Serum CA724, DKK1, and TK1 in Diagnosis of Gastric Cancer

**DOI:** 10.1155/2022/6941748

**Published:** 2022-10-14

**Authors:** Yu Wang, Dongqing Cui, Di Li, Dan Li, Honglei Wang, Yu Wu

**Affiliations:** ^1^Department of Gastrointestinal Surgery, Integrated Chinese and Western Medicine Hospital, Tianjin University, Tianjin 300100, China; ^2^Tianjin Key Laboratory of Acute Abdomen Disease Associated Organ Injury and ITCWM Repair, Tianjin 300100, China; ^3^NHC Key Laboratory of Hormones and Development, Chu Hsien-I Memorial Hospital and Tianjin Institute of Endocrinology, Tianjin Medical University, Tianjin 300134, China; ^4^Tianjin Key Laboratory of Metabolic Diseases, Tianjin Medical University, Tianjin 300134, China

## Abstract

**Objective:**

To explore the clinical value on combined detection of serum carbohydrate antigen 724 (CA724), secreted protein dickkopf-1 (DKK1), and thymidine kinase 1 (TK1) in the diagnosis of gastric cancer (GC).

**Methods:**

The clinical data of 63 GC patients (GC group) and 54 patients with benign gastric lesions (control group) admitted to Zhu Xianyi Memorial Hospital of Tianjin Medical University from June 2020 to June 2021 were retrospectively analyzed. The levels of serum CA724, DKK1, and TK1 in the two groups were detected by electrochemiluminescence instrument, enzyme-linked immunosorbent assay, and enhanced chemiluminescence. The diagnostic efficacy of single detection and combined detection was analyzed by drawing the receiver operating characteristic (ROC) curve.

**Results:**

Compared with the control group, the serological indexes of patients in GC group were markedly higher (*P* < 0.001). ROC curve analysis showed that the areas under the curve of serum CA724, DKK1, TK1, and combined detection in the diagnosis of GC were 0.849, 0.754, 0.685, and 0.923, respectively; and the sensitivity and specificity of their combined detection were higher than those of separate detection.

**Conclusion:**

The levels of serum CA724, DKK1, and TK1 were highly expressed in GC patients, with a higher diagnostic value for GC in their combined detection, which can effectively screen and assist the diagnosis of GC.

## 1. Introduction

Gastric cancer (GC) is a common tumor disease in digestive tract and one of the most common cancers in the world. In 2018, there were more than 1 million new cases and about 783,000 dead cases worldwide. Southeast Asia is a high-incidence area of GC in the world, and the prevention and control of GC in China are also severe [1]. Patients had no specificity in early clinical symptoms of patients, so it is often confused with gastritis or gastric ulcer in clinical diagnostic process [2]. Although there are many examination methods for this tumor disease, such as gastroscopy, X-ray examination, and CT scanning, the detection rate of early GC is still low, and most patients are already in the middle and late stages when the diagnosis seriously affects the clinical therapeutic effect [3], so that the accurate diagnosis of early GC has become the focus of attention in clinic. With the continuous progress of molecular biology technique, serum tumor markers have been gradually applied to the prediction of benign and malignant tumors. Studies have found that [4] the abnormal expression of human serum tumor markers occurs earlier than clinical symptoms. Some studies have also found [5] that CA724 in the diagnosis of positive rate in GC is higher than that of other tumor markers, which can be used as an important serum factor to judge the process of GC and clinical efficacy. DKK can regulate intracellular signal transduction by binding to its corresponding receptors, thereby determining the characteristics such as cellular differentiation, survival, and apoptosis, with an important effect in tumorigenesis [6, 7]. TK1 is a kinase related to cell proliferation, which plays a positive role in evaluation of cellular proliferating activity. The literature report has pointed out that [8] serum CA724 has an abnormal expression in early GC. Based on these, this study detected the serological indexes of GC and patients with benign gastric diseases and analyzed the diagnostic value of indexes and combined detection, so as to provide more references for choosing the treatment plan of GC.

## 2. Materials and Methods

### 2.1. General Information

63 GC patients (GC group) and 54 patients with benign gastric lesions (control group) admitted in Zhu Xianyi Memorial Hospital of Tianjin Medical University from June 2020 to June 2021 were selected as the study subjects for the retrospective analysis. This study has been approved by the Ethics Committee of Zhu Xianyi Memorial Hospital of Tianjin Medical University (approval no. 20200413) and was in line with the Declaration of Helsinki (2013) [9].

### 2.2. Inclusion and Exclusion Criteria

Inclusion criteria. (1) All patients in GC group were confirmed by surgical pathology, with the first onset. (2) Patients did not receive the chemotherapy or immunotherapy before admission. (3) Patients with benign gastric lesions were diagnosed by gastroscope and pathological examination.

Exclusion criteria. (1) Patients with other malignant tumors; (2) female patients in pregnancy or lactation period; and (3) patients with immune system disease or hematological disease.

### 2.3. Detection Method

Fasting elbow vein blood (3 ml) of all study subjects was obtained in the morning of admission, and the physiological saline was added to dilute the samples. After coagulation, the blood was centrifuged at 3000 rev/min for 5 min to separate the serum, and then, the clear supernatant was stored in a refrigerator at -80°C for examination. The CA724 level (reference range of 0-6.9 U/ml) in serum sample was detected using an electrochemiluminescence instrument (manufacturer: Shanghai Mojin Medical Instrument Co., Ltd.; model: Roche cobas e411), the serum DKK1 level (reference range of 0-3.3 ng/ml) was detected by enzyme-linked immunosorbent assay (kit purchased from Shanghai Fusheng Industry Co., Ltd.), and the enhanced chemiluminescence (kit purchased from Shanghai Toscience Biotechnology Co., Ltd.) was used to detect the TK1 level (reference range of 0-75.0 pmol/ml). All operations were strictly in accordance with the kit instruction.

### 2.4. Statistical Method

The SPSS 26.0 was used for the data analysis, the enumeration data such as gender and measurement data such as age and BMI value were indicated by (*n* (%)) and (^−^*x* ± *s*), and the comparisons between the two groups were tested by *x*^2^ and independent sample *t* test, respectively. The efficacy of separate detection of serum CA724, DKK, and TK1 and combined detection under the parallel structure model in the diagnosis of GC was analyzed using the ROC curve to obtain the sensitivity and specificity of diagnosis. *P* < 0.05 indicated a statistic significance.

## 3. Results

### 3.1. Comparison of Clinical Data between the Two Groups

There was no significant difference in clinical data such as age, WBC values, MCV values, and educational level (*P* > 0.05), see details in Table 1.

### 3.2. Comparison of Serological Indexes between the Two Groups

Compared with control group, the serological indexes in GC group were markedly higher (*P* < 0.001), see details in Table 2.

### 3.3. Diagnostic Efficacy of Serum CA724, DKK1, TK1, and Combined Detection in GC

**Figure 1:**
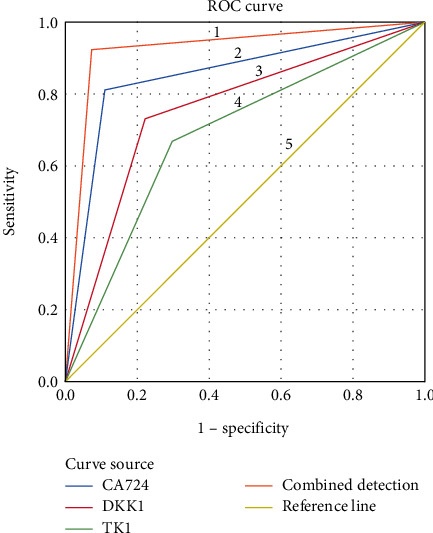
ROC curve of serum CA724, DKK1, TK1, and combined detection in the diagnosis of GC. Notes. 1 represented the ROC curve of combined detection for the diagnosis of GC, 2 represented the ROC curve of CA724 for the diagnosis of GC, 3 represented the ROC curve of DKK1 for the diagnosis of GC, 4 represented the ROC curve of TK1 for the diagnosis of GC, and 5 represented the reference line.

See Table 3 and Figure 1 for details.

## 4. Discussion

GC, as a malignant tumor originating from gastric epithelium, is a common tumor disease in digestive system, with a higher incidence [10], and it is classified as human group 1 carcinogen by the International Agency for Research on Cancer in the World Health Organization (WHO) [11]. There are many incidence positions of GC, such as curvatura ventriculi minor and gastric antrum, which often has no obvious symptom in the early stage of this disease and is easy to be ignored. Therefore, the disease has entered the middle and late stages when patients are diagnosed, which is not conducive to the treatment of disease. Although pathological biopsy under gastroscopy is recognized as the gold standard for the diagnosis of GC, it is not widely used in clinic due to the invasive operation [12, 13], so that the diagnosis of this disease has attracted the attention of many physicians in oncology department and patients.

With the progress of medical diagnosis technology, the advantages of serum tumor markers with high specificity and sensitivity and convenient detection in the differential diagnosis of GC have become the focus of clinical study [8]. Some studies [14] have confirmed that the combined detection of serum CA724 and CA19-9 has high specificity and sensitivity in the diagnosis of gastric stromal tumor, and studies have also reported [15] that serum DKK1 has an obviously high expression in patients with primary liver cancer. However, the sensitivity and specificity of detection with single indicator are low, which cannot meet the clinical needs due to the diversity and complexity of biological characteristics in tumor cells [16, 17]. Therefore, it is very important to combine different serum tumor markers for early diagnosis and treatment of GC patients to perform the combined detection of multiple targets. However, the value on combined detection of serum CA724, DKK1, and TK1 in the diagnosis of GC is not clear. In this study, it was found that the levels of serum CA724, DKK1, and TK1 in GC group were higher than those in control group by detecting the serological indexes of GC group and control group (*P* < 0.001). The putative reasons are as follows. CA724 is mucinoid glycoprotein polymer recognized by two monoclonal antibodies of cc49 and b72.3, with an abnormal increase in various gastrointestinal tumors and ovarian cancer [18]. DKK1 has been proved to be a new therapeutic target for lung cancer and esophageal cancer and plays an important role in the pathogenesis and development of gastric cancer, cervical cancer, and other malignant tumors [19]. The Wnt/*β*-catenin signaling pathway is inhibited due to the changes of *β*-catenin in GC patients, resulting in a high expression of DKK1. DKK1 is a secreted protein that can be secreted into the blood, and the serum DKK1 level will be significantly elevated with its increased expression in gastric cancerous tissues [20]. As a marker of abnormal cell proliferation, the content of TK1 is positively correlated with the situation of DNA synthesis in the body. Clinical studies have found [21] that the content of TK1 in nonproliferative cells is extremely low, which is involved in cell proliferation and plays an important role in the pyrimidine salvage pathway. Therefore, TK1 concentration will increase with the lesion growth, disease progression, and distant metastasis.

Although most clinical studies have suggested that patients with malignant tumors have an increase of tumor markers, there are still some factors that can affect the diagnosis and judgment. As previously reported [22], CA724 was significantly increased in 3.5% of healthy adults and 6.7% of patients with gastrointestinal diseases, and the secretion of DKK1 can also be affected by other factors and liver metabolism after entering the blood, which are the reasons for missed diagnosis and misdiagnosis of tumor markers. In addition, a single test for each serological marker was conducted in this study, which can easily lead to inaccurate detecting results, so that multiple measurements can be performed when conditions permit to obtain more accurate detecting results.

The ROC curve for the diagnosis of GC was constructed in order to accurately and comprehensively evaluate the value of each serological index in the diagnosis of GC. The results showed that the areas under the curve of CA724, DKK1, and TK1 for the diagnosis of GC were 0.849, 0.754, and 0.685, respectively, suggesting that each serological index has certain diagnostic value for GC, with a high missed diagnosis and misdiagnosis. At present, the combined diagnosis with multiple indicators is often used in clinic, which can improve the diagnostic efficiency of diseases to a certain extent [23, 24]. In this study, the diagnosis status of GC by single and combined diagnostic methods was analyzed, and the results showed that compared with single diagnosis, the area under the curve of the combined diagnosis was the highest, suggesting that the combined detection with multiple indicators could improve the diagnostic efficiency of GC. Therefore, the combined detection has the highest diagnostic value for GC, which can make up for the shortcomings of insufficient sensitivity or specificity of single index, so as to provide more accurate information for clinical practice. Compared with the previous serological detection tests in clinic, the results of this study confirmed that this scheme had higher diagnostic efficiency, and the detection method was convenient and fast, so as to better make up for the shortcomings of previous diagnosis, which is the promotion and optimization of previous studies.

## 5. Conclusion

The detection of tumor markers has an important significance for the diagnosis of GC in clinic, while the detection of single indicator has limitations in specificity and sensitivity, and the combined detection with different indicators can improve the diagnostic efficiency through complementary advantages, which can provide more evidence-based proofs for the treatment protocol of subsequent disease. However, the detection of serum tumor markers has its unique advantages, but also has some defects, so that it is still the focus of future studies to find ideal tumor markers related to GC and explore new detection methods.

## Figures and Tables

**Table 1:** Comparison of clinical data between the two groups.

Projects	GC group (*n* = 63)	Control group (*n* = 54)	*X* ^2^/*t*	*P*
Gender				
Male/female	35/28	30/24	0.000	1.000
Average age (mean ± SD, years)	48.92 ± 8.94	47.41 ± 8.18	0.947	0.346
BMI (mean ± SD, kg/m^2^)	21.36 ± 1.35	21.61 ± 1.33	1.005	0.317
Types of GC		/	/	/
Diffuse pattern	29 (46.03)	/		
Intestinal type	34 (53.97)	/		
TNM staging			/	/
Stage I	32 (50.79)	/		
Stage II	17 (26.98)	/		
Stage III	8 (12.70)	/		
Stage IV	6 (9.52)	/		
WBC (mean ± SD, ×10^9^/L)	6.01 ± 1.55	6.31 ± 1.53	1.258	0.223
HCT (mean ± SD, %)	0.39 ± 0.04	0.40 ± 0.06	1.537	0.097
MCV (mean ± SD, fl)	87.94 ± 5.21	86.57 ± 5.26	1.644	0.108
Educational level				
College and above	5 (7.94)	4 (7.41)	0.012	0.915
Senior high school	17 (26.98)	13 (24.07)	0.129	0.719
Junior high school	20 (31.75)	15 (27.78)	0.218	0.640
Primary school	12 (19.05)	18 (33.33)	3.112	0.078
Illiteracy	9 (14.29)	4 (7.41)	1.393	0.238
Place of residence (*n* (%))			0.030	0.863
Town	27 (42.86)	24 (44.44)		
Countryside	36 (57.14)	30 (55.56)		

**Table 2:** Comparison of serological indexes between the two groups (^−^*X* ± *S*).

Groups	*n*	CA724 (U/ml)	DKK1 (ng/ml)	TK1 (pmol/ml)
GC group	63	24.91 ± 2.01	4.56 ± 0.36	104.64 ± 11.48
Control group	54	4.05 ± 1.20	3.03 ± 0.40	66.36 ± 4.65
*t*		66.726	21.771	22.933
*P*		<0.001	<0.001	<0.001

**Table 3:** Diagnostic efficacy of serum CA724, DKK1, TK1, and combined detection in GC.

Diagnosis methods	Specificity	Sensitivity	Area under curve	Positive predictive value	Negative prediction value	Asymptotic 95% confidence interval
CA724	80.00%	89.47%	0.849	80.95%	88.89%	0.774-0.924
DKK1	71.19%	79.31%	0.754	73.02%	77.78%	0.663-0.845
TK1	64.41%	72.41%	0.685	66.67%	70.37%	0.587-0.783
Combined detection	90.91%	93.55%	0.923	92.06%	92.59%	0.867-0.979

## Data Availability

Data to support the findings of this study is available on reasonable request from the corresponding author.
